# The association between social position and self-rated health in 10 deprived neighbourhoods

**DOI:** 10.1186/s12889-015-1377-2

**Published:** 2015-01-21

**Authors:** Carsten Kronborg Bak, Pernille Tanggaard Andersen, Unni Dokkedal

**Affiliations:** Department of Health Science and Technology, Unit of Epidemiology and Public Health, Niels Jernes Vej 14, 9220 Aalborg, Denmark; Unit of Health Promotion, Department of Public Health, University of Southern Denmark, Niels Bohrs Vej 9, 6700 Esbjerg, Denmark; Unit of Epidemiologym Biostatistics and Biodemography, Department of Public Health, University of Southern Denmark, J.B. Winsløwsvej 9B, 5000 Odense, Denmark

**Keywords:** Self-rated health, Social position, Deprived neighbourhood, Index for life resources

## Abstract

**Background:**

A number of studies have shown that poor self-rated health is more prevalent among people in poor, socially disadvantaged positions. The aim of the present study was to investigate the association between self-rated health and social position in 10 deprived neighbourhoods.

**Methods:**

A stratified random sample of 7,934 households was selected. Of these, 641 were excluded from the study because the residents had moved, died, or were otherwise unavailable. Of the net sample of 7,293 individuals, 1,464 refused to participate, 885 were not at home, and 373 did not participate for other reasons, resulting in an average response rate of 62.7%. Multiple logistic regression models were used to estimate the associations between the number of life resources and the odds of self-rated health and also between the type of neighbourhood and the odds of self-rated health.

**Results:**

The analysis shows that the number of life resources is significantly associated with having poor/very poor self-rated health for both genders. The results clearly suggest that the more life resources that an individual has, the lower the risk is of that individual reporting poor/very poor health.

**Conclusions:**

The results show a strong association between residents’ number of life resources and their self-rated health. In particular, residents in deprived rural neighbourhoods have much better self-rated health than do residents in deprived urban neighbourhoods, but further studies are needed to explain these urban/rural differences and to determine how they influence health.

## Background

Self-rated health is an important measure of a person’s general health status [1,2]. A number of studies have shown that poor self-rated health is more prevalent among people in poor, socially disadvantaged positions [2]. In particular, the results of multilevel studies have shown that the residents of deprived neighbourhoods are more likely to rate their health as fair or poor compared with residents of more affluent neighbourhoods [3,4]. However, the focus in this study is only on deprived neighbourhoods.

Independent of individual characteristics, where people live has a substantial influence on health. Indeed, neighbourhood effects operate through the availability and accessibility of health services, infrastructure, attitudes towards health and behaviours, and social support [5-8].

The association between neighbourhood concentrations of disadvantage and poor health outcomes can be explained by theories of social disorganisation [9], which suggest that dimensions of community structure, including poverty, unemployment, and residential instability, result in a lack of necessary health-promoting infrastructure, limited material resources, and reduced health-related collective efficacy [8-13]. Stress levels have also been found to be higher in deprived neighbourhoods compared with wealthier residential areas [6,7]. Living in disadvantaged neighbourhoods appears to increase the exposure to stress and to sustain chronic stress due to limited services, poor infrastructure and a lack of social support, with worse self-reported health and more chronic conditions than in residents of more advantaged neighbourhoods [14,15].

Studying and fully understanding the association between an individual’s social status/position in society and his/her health are complicated endeavours [16]. Social position has often been measured using income, occupation, and education as single indicators of socioeconomic status [4], but which indicators are the best predictors of social position is still a topic of ongoing discussion among researchers [17].

It is reasonable, however, to assume that social position cannot be measured using a single variable because it is a concept that includes several dimensions [17-19]. In fact, the results of studies of the association between social position and self-rated health have been inconsistent and contradictory. Differences in morbidity and mortality between socioeconomic groups have been observed in many studies [20-22]. Additionally, a review of income inequality and population health showed substantial differences in findings depending on whether inequality was measured in large or small-scale areas [23]. Conversely, a minority of studies have concluded that inequalities do not have any implications for population health [24,25]. These differences in findings and conclusions may be related to difficulties in measuring socioeconomic position. These difficulties include the fact that health conditions change over the course of life and that health can be influenced by individual behaviour [26]. Therefore, we assume that these results indicate differences in the conceptualisation and measurement of social position [17].

Lynch and Kaplan [26] suggest a classification that uses three major sociological traditions that have influenced the measurement and understanding of social position: (1) the functionalist tradition, which argues that modern, complex societies require stratification into positions that are more or less valuable to society, and typical measurements of social position focus on occupational prestige; (2) the Weberian approach, which focusses on individual characteristics that influence an individual’s life opportunities, and typical measurements of social position include individual resources, such as education, income, and wealth; and (3) the (neo-)Marxian approach, which focusses on social structure, considered as a macro system for the allocation of life chances, and typical measurements focus on characteristics of the social structure, primarily including occupation or occupational social class [17].

In a previous study [19] of one deprived urban neighbourhood we used a resource index to measure social position. This study was inspired by the Weberian approach and focused on using individual resources to measure social position. We believe that an index can provide a better understanding of the complexity of the effect of social position on health by including more socioeconomic indicators in the same index [27]. The results of this study showed a strong association between the residents’ number of life resources and their self-rated health.

The starting point regarded social position from a generic resource perspective, whereby resource allocation within a population is assumed to influence an individual’s living conditions in terms of aiding health and well-being, among other aspects.

The resource perspective is closely connected to the Nordic welfare model. This model is centred on resource allocation within the population as a whole and focusses on resource development rather than economic redistribution [28-31]. The resource perspective is based on the idea that an individual acts with self-defined goals in mind but is limited in his/her ability to achieve these goals using the available resources [30,32].

Using a resource index to measure social position has a number of advantages [33,34]. In contrast to traditional categorisations based on occupational positions, a resource index for social position allows individuals outside the labour market to be positioned alongside working individuals. In addition, an index can include information on family structure and resources within a family (e.g., economic deprivation), which constitute important aspects of social position. A further advantage is that an index measures social position on a continuum, which provides more individual measures than does a set of rigid social position categories [35]. Finally, individuals are evaluated based on their total amount of non-prioritised resources, allowing the possession of one resource to compensate for a lack of another.

### Aim of this study

The aim of this study was to investigate the association between self-rated health and social position in 10 deprived neighbourhoods for further testing of the life resource index and to discuss the strengths and weaknesses of using the index as a measure of social position in deprived neighbourhoods.

## Method

### Materials

Data were provided by the Danish National Board of Health. These data were collected from January 3 to March 8, 2011, through surveys conducted in 10 socially deprived neighbourhoods located in 10 municipalities in Denmark. The data collection was performed by the Danish National Centre for Social Research, which is a sector research institution under the Danish Ministry of Social Affairs. Prior to the data collection, information about the survey was sent by postal letter in Danish, English, Turkish, Arabian, and Serbo-Croatian, and this information included a contact telephone number.

The survey was completed by telephone and face-to-face interviews.

The results in this article are based on a secondary analysis of these survey data.

“Deprived neighbourhood” is defined here as a geographically bounded area with a high proportion of adults outside the labour market, including people receiving social benefits, disability benefits or sickness benefits and people with low income, low education, or low-paid jobs. There are also often high proportions of non-Western immigrants and single parents in deprived neighbourhoods. Several of the included neighbourhoods are on the Danish government’s ghetto list, whereas others have certain (but not all) characteristics of a ghetto.

The Danish government’s classification of an area as a ghetto requires that it meets at least two of three criteria: a proportion of non-Western immigrants exceeding 50% of the proportion of 18- to 64-year-old adults, a number of adults outside the labour market exceeding 40%, and a number of convicts exceeding 270 persons per 10,000 persons in the neighbourhood [36]. A total of 21,605 persons older than 17 years live in the 10 neighbourhoods studied, 13% of whom receive social benefits, disability benefits, or sickness benefits and 55% of whom low education levels (i.e., a low graduation rate from secondary school or compulsory education). An additional 11% of individuals older than age 17 are single parents, and 19% are immigrants.

We distinguish between ghetto areas and non-ghetto areas because we assume that an accumulation of disadvantage will be more present in ghetto areas.

Two of the deprived neighbourhoods in this study differ from the other areas by being located in peripheral/rural areas. These areas are characterised by a lower population density and a majority of single-family houses compared with the other eight urban areas.

**Figure 1:**
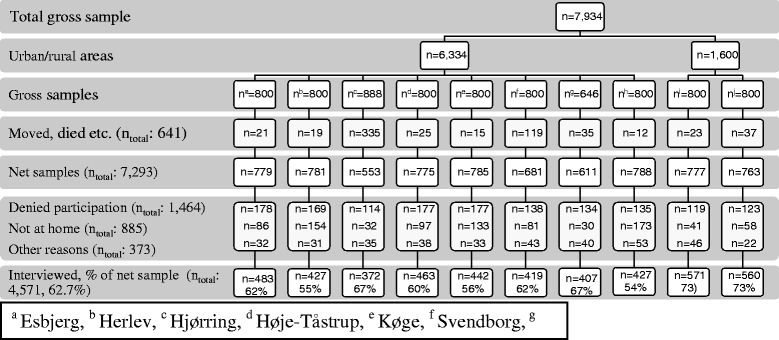
**Sampling scheme and participation in the study.** Figure 1 illustrates the sampling of participants in the study. The target group was defined as individuals above the age of 17 living in the neighbourhoods. For the current study, a stratified random sample of 7,934 households was selected. Of the 7,934 households, 641 were excluded from the study, as the residents had moved, died, or were otherwise unavailable. One person from each of the remaining households was selected, and quota sampling with respect to gender and age was used as the sampling procedure. Of the net sample of 7,293 individuals, 1,464 refused to participate, 885 were not at home, and 373 did not participate for other reasons, resulting in an average response rate of 62.7%.

The target group was defined as individuals older than age 17 living in the studied neighbourhoods. For the present study, a stratified random sample of 7,934 households was selected (Figure 1). Of the 7,934 households, 641 were excluded from the study, as the residents had moved, died, or were otherwise unavailable. One person from each of the remaining households was selected, and a quota sampling procedure with respect to gender and age was used. Of the 7,293 remaining individuals, 1,464 refused to participate, 885 were not at home, and 373 did not participate for other reasons (we do not have specific information about the reasons), resulting in an average response rate of 62.7%.

The survey was conducted primarily through telephone interviews and secondarily via personal interviews to increase the response rate. Whether the participants were interviewed by telephone or in person was not documented.

### Measures

#### Questionnaire development

The questionnaire used in the study was constructed according to standards based on existing research [37] and was designed to collect information on health indicators, health behaviours, and social factors. The 62-question questionnaire was specifically developed to form the basis for health-related intervention projects in the neighbourhoods [38].

#### Self-rated health

The outcome variable was the widely used self-rated health measure. The respondents were asked “How would you describe your health in general?”, with the response categories of “very good”, “good”, “neither good nor bad”, “bad” and “very bad”. The scale was dichotomised, with 1 representing bad and very bad health and 0 representing neither good nor bad health as well as good and very good health.

#### Social position

To measure social position, we used an index for life resources that was used in previous studies of social position and self-rated health in Denmark [19,32]. The index is a resource index that includes both socioeconomic factors, including education, job, and income, and family conditions, such as marital status and having children [35]. The index is based on a formative measurement model in which the theoretical variable (i.e., social position) is caused by the resource variables included in the index, as opposed to a reflective measurement model, in which the opposite causal relationship between the theoretical variable and the indicators is assumed.

#### Index for life resources

We used a version of the index that is slightly adjusted compared with the one used in a previous study in one deprived neighbourhood [19]. We omitted job type (white-collar worker or self-employed, yes/no) because the questionnaire did not provide the needed information. The following variables are included in the index for life resources: living with others, children, education, occupational income, disposable income, and economic deprivation (Table 1).

**Table 1:** **Variables applied in the life resources index**

	**Resource**	**Lacking resource**
Living with others	Yes	No
Has children	Yes	No
Has studied beyond primary school	Yes	No
Has occupational income	Yes	No
Has monthly disposable income ≥ DKK 4,000	Yes	No
Suffers from no economic deprivation	Yes	No

The median minimum disposable income was DKK 4,000 per month after fixed expenses (approximately USD 700/GBP 450 in 2011 currency) in the study population, and this income was used to compare the economically worse-off and better-off groups of residents in the neighbourhoods. Economic deprivation is defined here as a situation in which an individual or a family has been unable to pay their bills; afford dental check-ups; or buy birthday presents, medicine, clothes, and other items for economic reasons over the last year.

The index for life resources assigns values from zero to six. A low-index value indicates an individual with few resources, and a high value indicates an individual with many resources. We grouped the values into an ordinal variable with the categories 0–1, 2, 3, 4, and 5–6 resources.

#### Ethnicity

Ethnicity was established by the questionnaire by asking respondents questions about their birth countries and those of their parents. The categorisation of the participants into native Danes, people with Western backgrounds and those with non-Western backgrounds was based on a slightly modified version of the Statistics Denmark’s classification of the population into three groups (immigrants, descendants of immigrants, and native Danes) [39]. Statistics Denmark specifically defines immigrants as people who are foreign born and whose parents are foreign born or have foreign citizenships, and descendants of immigrants are defined as people born in Denmark and whose parents are either foreign born or hold foreign citizenship. In contrast, native Danes are defined as people who have at least one parent who is a Danish citizen and who was born in Denmark.

Because of the very small numbers of descendants, immigrants and descendants were collapsed into one group and then categorised as people with a Western or non-Western background. Western countries were defined as the EU countries, other Nordic countries, and other Western countries (e.g., the USA and Canada).

### Statistical analysis

Preliminary analyses were performed and included frequency tables, cross-tabulations, and chi-squared tests.

Multiple logistic regression models were used to estimate the associations between the number of life resources and the odds of self-rated health. As two of the ten neighbourhoods are rural areas, we wanted to investigate whether rurality has an independent effect on self-rated health in addition to one's resources, and thus we included a dummy variable for urban/ rural differences in the analysis.

We further tested for effect modification of the associations by gender. Adjustments were made for age, ethnicity, and the type of neighbourhood, which were considered as the main relevant confounders.

In this study, p-values < 0.05 were regarded as statistically significant. The statistical analyses were performed using STATA® version 12.0 (Stata Corp LP, 4905 Lakeway Drive, TX, USA).

## Results

The demographic characteristics of the sample are shown in Table 2. The mean ages of the participants were 48.8 years for males and 49.8 years for females. Approximately 17% of the male participants and 13% of the females had non-Western backgrounds. The majority of the non-Western residents came from Turkey (32%), Iraq (8%), Vietnam (5%), Iran (4%), Bosnia (4%), and Kosovo (4%). Approximately 75% of the participants lived in urban neighbourhoods, whereas 25% lived in rural neighbourhoods.

**Table 2:** **Characteristics of the study population**

		**Males (n = 2,109)**		**Females (n = 2,462)**	
Age (mean, SD)		48.8	(17.2)	49.8	(17.9)
Ethnicity (n,%)	Native Danes	1,718	(81.5)	2,081	(84.6)
	Other Western background	38	(1.8)	59	(2.4)
	Non-Western background	351	(16.7)	320	(13.0)
Life resources (n,%)	0-1	342	(18.4)	351	(17.0)
	2	333	(21.3)	395	(19.2)
	3	381	(21.8)	453	(22.0)
	4	372	(19.0)	418	(20.3)
	5-6	321	(19.6)	445	(21.6)
Type of neighbourhood (n,%)	Urban	1,572	(74.5)	1,868	(75.9)
	Rural	537	(25.5)	594	(24.1)
Self-rated health (n,%))	Very good	471	(22.4)	532	(21.7)
	Good	895	(42.6)	1,039	(42.3)
	Neither good nor bad	403	(19.2)	451	(18.4)
	Bad	248	(11.8)	349	(14.2)
	Very bad	86	(4.1)	84	(3.4)

For both genders, approximately 65% of participants reported having good/very good self-rated health, whereas 16% reported having poor/very poor health. There was certain evidence supporting modification of the association between the number of life resources and the odds of self-rated health by gender (p = 0.0356). Consequently, all subsequent analyses were stratified by gender.

Table 3 shows that the number of life resources is significantly associated with having poor/very poor self-rated health for both genders. The results clearly suggest that the more life resources that an individual has, the lower the risk is of that individual reporting poor/very poor health. More specifically, a male with no or only one life resource has an approximately 6 times greater risk of reporting poor/very poor health compared with a male with many life resources (5–6), whereas a female with no or only one life resource has a 4 times greater risk of reporting poor/very poor health compared with a female with many life resources.

**Table 3:** **Associations between life resources and poor/very poor self-rated health**

**Odds ratio (95% confidence interval)**								
**Life resources**	**N**	**Males** ^**a**^	**N**	**Females** ^**a**^	**N**	**Males** ^**b**^	**N**	**Females** ^**b**^
0-1	341	7.98 (4.51-14.15)^**^	350	4.89 (3.14-7.61)^**^	341	6.48 (3.61-11.62)^**^	349	4.36 (2.77-6.85)^**^
2	333	4.50 (2.50-8.09)^**^	395	4.30 (2.78-6.65)^**^	332	3.63 (1.99-6.60)^**^	395	3.78 (2.43-5.88)^**^
3	381	2.63 (1.44-4.80)^**^	453	3.63 (2.36-5.59)^**^	381	2.11 (1.15-3.89)^*^	453	3.24 (2.09-5.01)^**^
4	372	1.84 (0.98-3.45)	417	1.36 (0.83-2.23)	372	1.66 (0.88-3.11)	417	1.27 (0.77-2.09)
5-6	321	Reference	445	Reference	320	Reference	445	Reference

^*^< 0.05.

^**^< 0.01.

^a^Adjusted for age.

^b^Adjusted for age, ethnicity, and the type of neighbourhood (urban or rural).

Table 4 shows that a male living in a rural neighbourhood has a 40% lower risk of reporting poor/very poor health than does a male living in an urban neighbourhood. The table also shows that a female living in a rural neighbourhood has a 26% lower risk of reporting poor/very poor health than does a female living in an urban neighbourhood.

**Table 4:** **Associations of the type of neighbourhood (urban or rural) with poor/very poor self-rated health**

**Odds ratio (95% confidence interval)**								
**Type of neighbourhood**	**N**	**Males** ^**a**^	**N**	**Females** ^**a**^	**N**	**Males** ^**b**^	**N**	**Females** ^**b**^
Urban	1,568	Reference	1,860	Reference	1,330	Reference	1,604	Reference
Rural	535	0.38 (0.27-0.52)**	594	0.54 (0.41-0.71)**	416	0.60 (0.40-0.89)*	455	0.74 (0.52-1.04)

^*^< 0.05.

^**^< 0.01.

^a^Adjusted for age.

^b^Adjusted for age, ethnicity and life resources.

## Discussion

The main purpose with this article was to further test the association between self-rated health and social position using an index for life resources with survey data from 10 deprived neighbourhoods in Denmark and to discuss its strengths and weaknesses.

To a certain extent, our findings are consistent with international research in this field. In particular, a number of multilevel studies have shown that residents of deprived neighbourhoods are more likely to rate their health as fair or poor [19] compared with, for example, residents of more affluent neighbourhoods [3,4].

Conversely, the findings from a study in the USA [40] were the opposite of our results, with residents of remote rural counties having the greatest odds of reporting bad health. The researchers of that study concluded that the significant differences in self-rated health between metropolitan residents and residents of rural areas can be entirely explained by a rural *structural disadvantage*, which concerns information of, for example, different county-level variables that tap concepts of social disorganisation and economic disadvantage, unemployment rates and poverty rates [40].

In contrast, our results are consistent with previous research [41] that demonstrated that the prevalence of all measures of poor health is indeed higher in deprived areas but is mainly attributable to individual socio-economic status (SES). We cannot, however, overlook the contextual effects; our results indicate that living in a rural neighbourhood is better for self-rated health than living in an urban neighbourhood is. As the data do not provide concrete explanations for this difference, additional analysis is needed.

Our results are also consistent with results obtained in a previous Danish study [32], even though the Larsen study differs from our study in certain areas. Our study examined 10 deprived neighbourhoods hosting a relatively large number of residents with few resources, whereas the Larsen study examined the general population in a county and did not include information about comparisons between people from deprived neighbourhoods with more affluent neighbourhoods. The resource index that we used in this study also includes economic variables that slightly differ from those of Larsen’s (e.g., disposable income and deprivation). Finally, we also tested for urban–rural differences, though this test was not the main purpose with the research. The test for urban–rural differences was not considered in Larsen’s 2003 study.

A study of how residents in 12 deprived urban neighbourhoods in Denmark evaluate their neighbourhood showed a strong connection between the residents’ perception of the reputation of their neighbourhood and their plans to move but that a number of other factors have great importance – e.g., dissatisfaction with social problems and crime – especially among residents with employment [42].

This issue is important when we attempt to interpret our results from 8 urban and 2 rural deprived neighbourhoods, which showed that residents in rural areas have a significantly lower risk of reporting poor/very poor health. We need to take into consideration that it is often the residents with the best life resources in these areas that choose to move out of these areas when their income increases, e.g., because they get a new job or a better paying job. This high mobility does not occur in rural areas.

We chose to include life resources that are perceived as important in our index to measure social position and have been validated in previous Danish studies [19,32], but these resources may have a different impact on residents in urban deprived neighbourhoods than on residents in rural areas.

There is a need for further research into the differences that exist between rural and urban areas with respect to life-generated resources and health. Public health interventions should address population heterogeneity and life resources, the specific factors relevant to health that emerge in specific circumstances.

### Study limitations

The resource index that we used is based on theoretical considerations. We also used this index in a previous study, along with data from one deprived urban neighbourhood [19], but the index still needs to be developed and validated in further studies. Additionally, the measure of self-rated health was based on a single-item questionnaire, which might have certain drawbacks compared with, for example, SF-36. However, the single-item questionnaire has been validated and used as a global measure of general health status in epidemiology and social science [19].

As for most studies based on survey data from questionnaires, our results should be interpreted cautiously because of the relatively large number of non-responders. Our 62.7% response rate, however, is highly consistent with rates in similar investigations. There are also further limitations to the data collection in this study. The survey was primarily conducted through telephone interviews; personal interviews were used secondarily to increase the response rate. However, which of the participants were interviewed by telephone and which were interviewed in person was not recorded.

Another limitation of this study is that data were only available from two rural areas, and most of the 671 non-Western respondents in the survey live in the deprived urban neighbourhoods. This fact makes it difficult to include analyses of ethnicity divided into subgroups when comparing between the urban and the rural neighbourhoods.

The aim of this study was to analyse the association between social position and self-rated health in 10 deprived neighbourhoods. However, we were only able to investigate the associations and did not analyse the importance of, for example, neighbourhood effects, which would have improved the results.

However, it could also be relevant to compare our findings with results from larger areas (on the municipality or regional level) or with results from more affluent areas [3], though our data did not provide this opportunity. Nonetheless, it would be relevant to address this in further research.

## Conclusions

In this study, we used a resource index to measure social position in 10 deprived neighbourhoods. To our knowledge, this is the first Danish study of the association between self-rated health and social position using survey data collected in deprived neighbourhoods.

The results show a strong association between the residents’ number of life resources and their self-rated health. Residents in rural neighbourhoods have much better self-rated health than do residents in deprived urban neighbourhoods. However, more research in Denmark is needed to explore the differences between urban and rural neighbourhoods and to learn how these differences (might) influence health, as most of the existing Danish studies have been based on national or regional/municipality-level data. Among the strengths of using data from specific neighbourhoods is the inclusion of vulnerable residents who seldom participate in national or regional studies.
